# Investigation of a Cost-effective and Durable Material for Containing Ballistic Gel in the Construction of Ultrasound Phantoms

**DOI:** 10.7759/cureus.5220

**Published:** 2019-07-24

**Authors:** Aaron Damon, William Clifton, Conrad Dove, Rachel Stein, Leslie V Simon

**Affiliations:** 1 Neurosurgery, Mayo Clinic, Jacksonville, USA; 2 Neurosurgery, Mayo Clinic, Rochester, USA; 3 Emergency Medicine / Medical Simulation, Mayo Clinic, Jacksonville, USA

**Keywords:** simulation, lumbar puncture, procedural skills, ultrasound, synthetic ballistical gel, 3d print, hdpe, marine starboard, medical education

## Abstract

There is significant variability in the realism, cost, and structural integrity of sonographic simulators available for use currently. A common material that is used for the production of sonographic simulators is synthetic ballistic gelatin, which requires a high melting temperature for molding. In this experiment, we investigated the structural integrity of high-density polyethylene (HDPE) when exposed to melted ballistics gel for the assimilation of a sonographic lumbar puncture simulator.

## Introduction

Medical simulation is a vital tool for procedural skills training and assessment [1]. Procedural simulation, in particular, has been shown to improve trainee ability and skill without added risk to live patients [2]. Task trainers are frequently utilized to enhance and assess procedural competency. Procedures involving cannulation of deep structures such as the spinal canal, major vessels, and other organs often utilize ultrasound guidance for insertion [3]. Sonographic task trainers or ultrasound phantoms allow learners to practice procedures under direct ultrasound guidance. There is significant variability in the realism, structural integrity, and simulated pathology of commercially available ultrasound phantoms. Cost is also variable, depending on the size and features of the simulator, and can often be a barrier to use. As a result, simulation specialists often seek to develop their own ultrasound phantoms to meet their needs. A common material used for “in-house” ultrasound phantom production is ballistic gel. Ballistic gel is low-cost and anechoic but has the disadvantage of a high melting temperature, which presents challenges in constructing simulators with large scales or complex shapes due to container compatibility. Polypropylene is a relatively flexible thermoplastic that is available for three-dimensional (3D) printing. Polypropylene has been previously studied for containing ballistics gel for simulator assembly [4]. High-density polypropylene (HDPE), also known as “marine starboard,” is a robust thermoplastic that is often used in the construction of waterproof vessels. Its high thermal resistance, relatively low cost, and ease of construction make it a suitable material for the creation of simulators that require the molding of melted ballistics gel. In this experiment, we compared the structural integrity of polypropylene to HDPE when exposed to melted ballistics gel for the assimilation of a sonographic lumbar puncture simulator.

## Technical report

**Figure 1:**
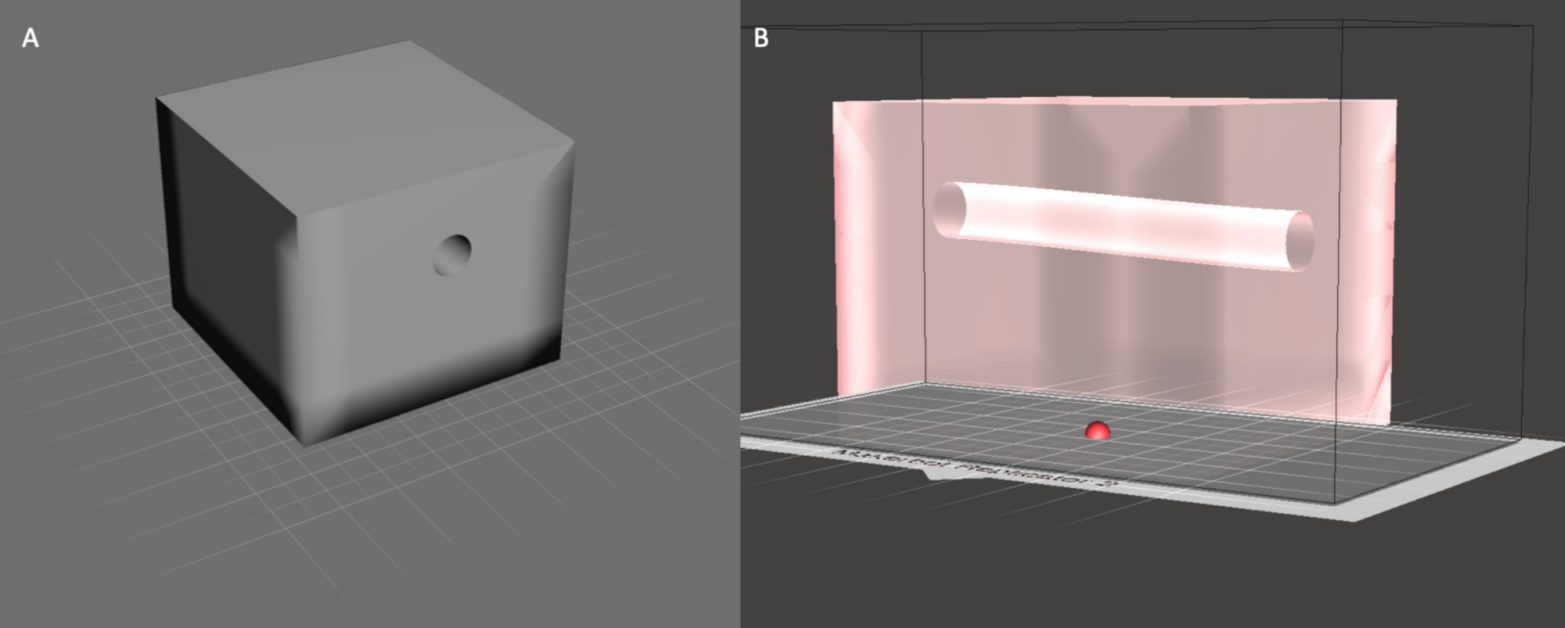
Stereolithography file design of polypropylene model (A). Computer-generated stereolithography file of the housing box for the lumbar puncture simulator. (B). Hollow projection demonstrating design and dimensions to accommodate the spine model for simulator assembly.

A sheet of HDPE that was 48 inches x 24 inches x ½ inches in dimension was acquired. The HDPE was cut with a standard table saw with a wood cutting blade. Three sections with dimensions of 13 inches in length and 5 inches in width were cut to create the side and bottom sections of the trainers. Two end caps with a width of 5 inches and a height of 4.5 inches were then cut on the table saw. A hand drill, standard wood screws, drill bits, and drivers were used to assemble the cut sections of the HDPE. A housing box for the lumbar puncture simulation was constructed successfully with dimensions of 13 inches x 5 inches x 5 inches to allow for the placement of a spine model within the container. As a control, a polypropylene container was designed to accommodate the desired structural components of the lumbar puncture simulator. The utilization of an Ultimaker 5S 3D printer (Utrecht, Netherlands) was planned to construct the polypropylene housing unit. Polypropylene filament (2.85 mm in diameter) was acquired in a 750-gram roll. An STL (stereolithography) file was created using open-access software (Meshmixer, Autodesk, California, US; 2017) of a rectangular, hollow unit with similar dimensional properties to the constructed HDPE container (see Figure 1). The STL file was sliced with Cura (v. 4.0; 2018) programming software (Ultimaker), with acceptable printer dimensions for production. In order to determine the material thermo-properties of polypropylene before undergoing time-intensive deposition modeling with the 3D printer, a commercially available polypropylene container was acquired for initial testing. Printing of the polypropylene STL file was planned if the initial material testing showed success in containing the liquid gel in order to conserve material and time expenditures.

**Figure 2:**
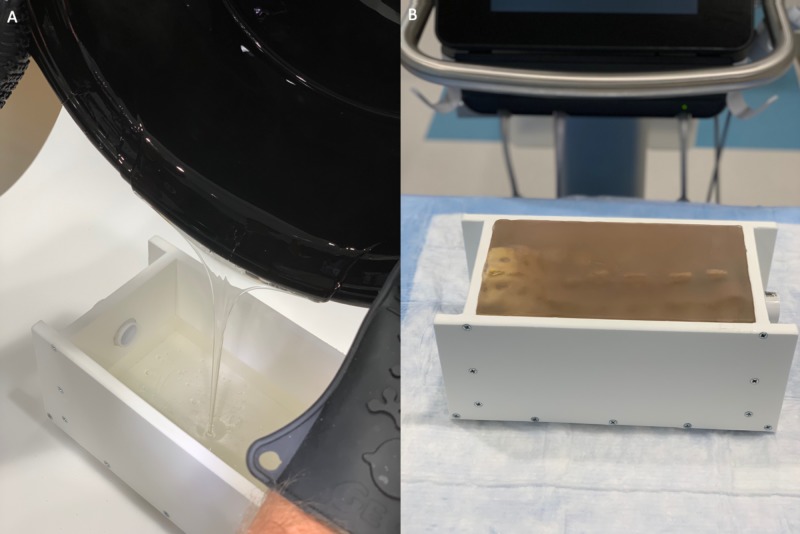
Pouring of melted ballistics gel into the HDPE container (A) Liquid gel is successfully contained within the walls of the simulator box. (B) The finished simulator with the inserted spine model for lumbar puncture simulation. HDPE: high-density polyethylene

**Figure 3:**
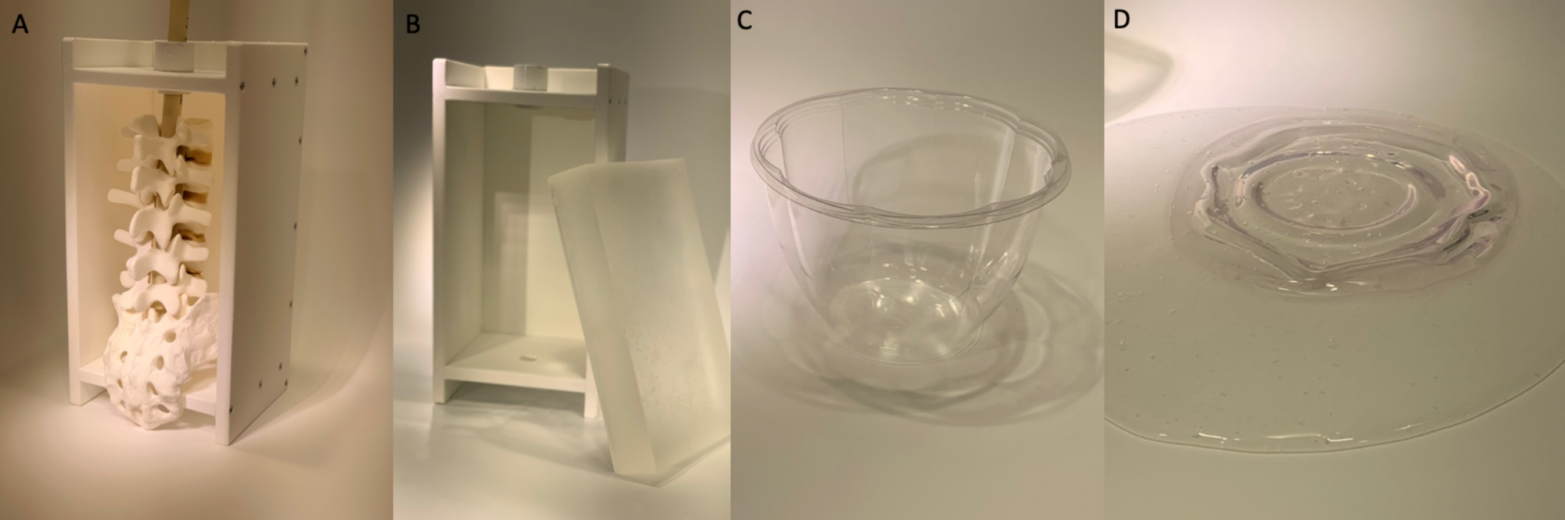
Results of HDPE and polypropylene comparison (A&B) The HDPE container tolerated the melted ballistics gelatin without deformation. (C&D) In contrast, the polypropylene container melted instantaneously upon contact with the gelatin. HDPE: high-density polyethylene

A 953-gram block of clear 20% ballistics gel was heated until the melting point at 115 degrees Celsius (C). Once the ballistics gelatin was completely liquified, a 20-ounce volume was poured into the HDPE container, and a similar volume was poured into the polypropylene container. Primary outcome measures were the ability of each container to properly contain the melted ballistics gelatin while maintaining its original form. If successfully maintained by the specific container material, the gel would be cooled at room temperature for a 12-hour period before quality assessment per manufactural protocol. Secondary outcome measures included a quality assessment of the gel through ultrasound to assess the identification of pertinent simulator structures. The visual results are depicted in Figure 2 and Figure 3. The melted ballistics gel was successfully poured into the HDPE container. The HDPE box retained its exact 13-inch x 5-inch dimensions and withstood the high temperature of the liquid gel without warping or breakage of the container walls. In comparison, the polypropylene container immediately melted and could not withstand the high temperatures of the melted gel. Due to the failure of initial material testing, the printing of the polypropylene container was deemed futile to construct the desired simulator.

**Figure 4:**
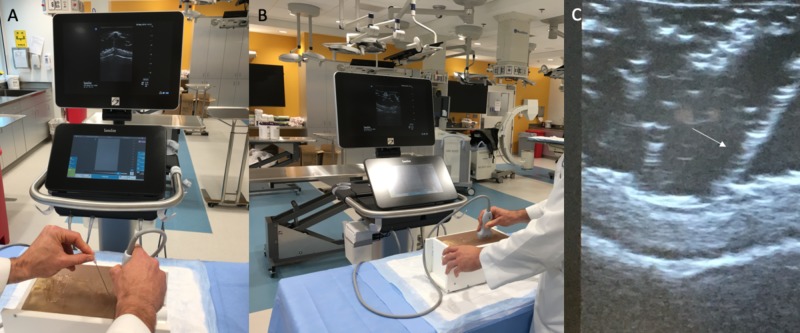
Sonographic properties of the constructed simulator (A&B). A surgical resident demonstrating the use of the assembled simulator that successfully shows (C) the position of the needle within the gelatin (white arrow). There were no barriers to sonography in the model for all participants.

The lumbar puncture simulator was then assembled within the HDPE container and tested. Thirteen (13) participants engaged in lumbar puncture simulation on the constructed apparatus. They were no instances of simulator breakage, leakage, or failure on multiple puncture attempts and manipulation. Sonographic images obtained using the lumbar puncture simulator were of high quality and adequately demonstrated needle placement for trainee instruction (see Figure 4).

## Discussion

Synthetic ballistic gel is commonly used for forensic ballistic analysis as well as medical simulation due to its thermal and mechanical stability and anechoic properties. Traditional container designs for pouring synthetic ballistic gel are typically composed of galvanized steel due to the high temperatures required to melt and mold the gel. Steel requires considerable resources to form complex shapes often required for medical simulation, including maintenance equipment and specialized facility equipment due to safety hazards when cutting the material. HDPE has robust material properties that can contain melted ballistic gelatin to create custom shapes. In our experiment, it markedly outperformed polypropylene in containing liquid gel, which has been previously used to construct sonographic simulators [4]. The structural integrity of constructed HDPE is an attractive feature when designing simulators to contain high volumes of other materials, such as our lumbar puncture simulator. Another advantageous feature is its high strength to density ratio that allows it to withstand higher temperatures than most polyethylene products. HDPE has material properties that can withstand temperatures of up to 135 degrees Celsius without softening. In addition, it also has an antimicrobial factor that resists staining and microbial growth, preventing rotting when wet. Our ultrasound phantom was constructed for less than $50 in structural material. Comparatively, other thermo-resistant materials, such as metal, require a much higher monetary expenditure for construction. A potential disadvantage of using HDPE is that it does require some effort with assembly, as well as standard woodworking tools to cut. While this requires some resources and time, it allows for complete customization and limitless possibilities for ultrasound model creation, as well as increased safety as compared to steel. 3D printing has been performed successfully for the design of complex simulation by our team, however, in this instance, the material properties of the chosen polymer could not withstand the high temperature of the melted ballistics gel. The balance between form and function is a fine line that must be navigated when choosing the simulator construction method and compositing material.

## Conclusions

This experiment showed considerable success in the use of high-density polyethylene sheets, also known as “marine starboard,” to contain ballistics gel in its liquid form during complex simulator construction as compared with polypropylene. To our knowledge, this is the first report of the successful use of HDPE to contain melted ballistics gel for ultrasoundable phantom construction. Our experiment showed that the low cost, safety profile, and durable material properties make HDPE an ideal material for the in-house production of these simulators.
